# Supplementary material for: Genetic and Evolutionary Analysis of Canine Coronavirus in Guangxi Province, China, for 2021–2024
Source: Vet Sci. 2024 Sep 26;11(10):456. doi: 10.3390/vetsci11100456 (PMC11512276; doi:10.3390/vetsci11100456)
Supplement: Supplementary file 1 [file vetsci-11-00456-s001.zip › vetsci-3189883-supplementary.pdf]

Supplementary Materials

Table S1 Information on Coronavirus Strains

Strain	Location	Code Letter	Date	Host	Gene Accession Number	Species
79-1146-CA-FCoV-II	USA	USA	1979	Feline	AY994055	α
171-CCoV-II	Germany	GER	1971	Canine	KC175339	α
DF-2-FCoV-II	USA	USA	1980	Feline	JQ408981	α
WSU-79-1683-FCoV-II	USA	USA	2011	Feline	JN634064	α
WH-1-TGEV	China	CHN	2010	Sus scrofa	HQ462571	α
virulent Purdue-TGEV	USA: Indiana	USA	1952	porcine	DQ811789	α
CB/05-CCoV-II	Italy	ITA	2005	Canine	KP981644	α
INSAVC-1-CCoV-II	UK	UK	1992	Canine	D13096	α
BGF10-CCoV-II	United Kingdom	UK	2002	Canine	AY342160	α
10/22-CCoV-II	United Kingdom	UK	2022	Canine	OX335534	α
UCD1-FCoV-I	United States	USA	1976	Feline	AB088222	α
KU-2-FCov-I	Japan	JPN	1994	Feline	D32044	α
Elmo/02-CCoV-I	Italy	ITA	2002	Canine	AY307020	α
23/03-CCoV-I	Italy	ITA	2002	Canine	AY307021	α
HCoV-NL63	China	CHN	2008	Homo sapiens	KM055650	α
HCoV-229E	China: Hong Kong	CHN	Jun, 2016	Homo sapiens	MT797716	α
CV777-PEDV	China	CHN	1994	swine	JN599150	α
BatCoV-HKU8	China: Hong Kong	CHN	2008	/	NC_010438	α
HKU9-1 BF_005I_BatCoV	China	CHN	2006	bat	NC_009021	β
HKU5_BatCoV	China	CHN	2006	bat	NC_009020	β
Wuhan-Hu-1_SARA-CoV-2	China	CHN	Dec, 2019	Homo sapiens	NC_045512	β
SARS-CoV-2-2019nCoV	Austria	AUT	Mar, 2021	Homo sapiens	ON112351	β
NDL_IPP01-HCoV-HKU1	The Netherlands	NL	2022	Homo sapiens	OR260091	β
Parker-RatCoV	USA	USA	2009	rat	NC_012936	β
3239-17-MHV	USA	USA	2011	/	JQ173883	β
7881-ECoV	China	CHN	2020	donkey	OM937885	β
UU-PHEV	The Netherlands	NL	Apr, 2015	pig	KY492680	β
KLF_HCoV-OC43.fasta-1	Kenya	KEN	Jan, 2018	Homo sapiens	MN026164	β
FRA-EPI-Caen-BCoV	France	FR	2003	bovine	KT318115	β
cattle_B277a-BCoV	China	CHN	Dec, 2021	Dairy Calves	OP866729	β
21032451-2CRCOV	China	CHN	Mar, 2021	dog	OQ351919	β
06-075-CRCoV	Japan	JPN	2007	dog	AB370269	β
SW1_BWCoV	USA	USA	2007	Delphinapterus leucas (beluga whale)	NC_010646	γ
TX-R-98_TCoV	USA	USA	1998	turkey	GU213202	γ
ATCC_TCoV	USA: Indiana	USA	2007	embryonated turkey eggs (21 day)	EU022526	γ
Beaudette-IBV	Singapore	SGP	2005	/	DQ001342	γ
HKU20-9243-WiCoV	China: Hong Kong	CHN	2008	wigeon	NC_016995	δ
HKU19-6918-NHCoV	China: Hong Kong	CHN	2007	night-heron	NC_016994	δ
HKU12-600-ThCoV	China: Hong Kong	CHN	Jan, 2007	grey-backed thrush	NC_011549	δ
HKU18-chu3-MRCoV	China: Hong Kong	CHN	2007	magpie-robin	NC_016993	δ
HKU17-6124-SPCoV	China: Hong Kong	CHN	2007	sparrow	NC_016992	δ
HKU21-8295-CMCoV	China: Hong Kong	CHN	2007	common moorhen	NC_016996	δ
HKU16-6847-WECoV	China: Hong Kong	CHN	2007	white-eye	NC_016991	δ
HKU11-796-BuCoV	China: Hong Kong	CHN	Jan, 2007	Chinese bulbul	FJ376620	δ
YRQ-PDCoV	China	CHN	2016	swine	OP566509	δ
F230-ALCCoV	China	CHN	2006	/	EF584908	δ

Table S2 Information on CCoV Gene Sequences Obtained in This Study

Name	Location	Date	Host	GenBank Accession Number		
				S gene	M gene	N gene
CCoV-CHN-2021-GXHCHB1221-1	Guangxi, China	Dec 21, 2021	Canine	PP583073	PP583138	PP583203
CCoV-CHN-2021-GXHCHB1221-2	Guangxi, China	Dec 21, 2021	Canine	PP583074	PP583139	PP583204
CCoV-CHN-2022-GXLZLC0220-3	Guangxi, China	Feb 20, 2022	Canine	PP583075	PP583140	PP583205
CCoV-CHN-2022-GXLZBD0304-4	Guangxi, China	Mar 4, 2022	Canine	PP583076	PP583141	PP583206
CCoV-CHN-2022-GXNNCL0509-5	Guangxi, China	May 9, 2022	Canine	PP583077	PP583142	PP583207
CCoV-CHN-2022-GXBSHJ0509-6	Guangxi, China	May 9, 2022	Canine	PP583078	PP583143	PP583208
CCoV-CHN-2022-GXBSHM0528-7	Guangxi, China	May 28, 2022	Canine	PP583079	PP583144	PP583209
CCoV-CHN-2022-GXLZXL0530-8	Guangxi, China	May 30, 2022	Canine	PP583080	PP583145	PP583210
CCoV-CHN-2022-GXBSHJ0611-9	Guangxi, China	Jun 11, 2022	Canine	PP583081	PP583146	PP583211
CCoV-CHN-2022-GXBSHM0615-10	Guangxi, China	Jun 15, 2022	Canine	PP583082	PP583147	PP583212
CCoV-CHN-2022-GXYLAC0803-11	Guangxi, China	Aug 3, 2022	Canine	PP583083	PP583148	PP583213
CCoV-CHN-2022-GXBSHM0824-12	Guangxi, China	Aug 24, 2022	Canine	PP583084	PP583149	PP583214
CCoV-CHN-2022-GXBSHM0915-13	Guangxi, China	Sep 15, 2022	Canine	PP583085	PP583150	PP583215
CCoV-CHN-2022-GXBSHM0915-14	Guangxi, China	Sep 15, 2022	Canine	PP583086	PP583151	PP583216
CCoV-CHN-2022-GXYLAC1004-15	Guangxi, China	Oct 4, 2022	Canine	PP583087	PP583152	PP583217
CCoV-CHN-2022-GXGLKB1028-16	Guangxi, China	Oct 28, 2022	Canine	PP583088	PP583153	PP583218
CCoV-CHN-2022-GXGLKB1030-17	Guangxi, China	Oct 30, 2022	Canine	PP583089	PP583154	PP583219
CCoV-CHN-2022-GXBSHJ1005-18	Guangxi, China	Oct 5, 2022	Canine	PP583090	PP583155	PP583220
CCoV-CHN-2022-GXBSHJ1007-19	Guangxi, China	Oct 7, 2022	Canine	PP583091	PP583156	PP583221
CCoV-CHN-2022-GXBSHJ1011-20	Guangxi, China	Oct 11, 2022	Canine	PP583092	PP583157	PP583222
CCoV-CHN-2022-GXBSHJ1016-D21	Guangxi, China	Oct 16, 2022	Canine	PP583093	PP583158	PP583223
CCoV-CHN-2022-GXBSHM1008-22	Guangxi, China	Oct 8, 2022	Canine	PP583094	PP583159	PP583224
CCoV-CHN-2022-GXGLBD1002-23	Guangxi, China	Oct 2, 2022	Canine	PP583095	PP583160	PP583225
CCoV-CHN-2022-GXBSPG1123-24	Guangxi, China	Nov 23, 2022	Canine	PP583096	PP583161	PP583226
CCoV-CHN-2022-GXYLAC1203-25	Guangxi, China	Dec 3, 2022	Canine	PP583097	PP583162	PP583227
CCoV-CHN-2022-GXBSHJ0711-26	Guangxi, China	Jul 11, 2022	Canine	PP583098	PP583163	PP583228
CCoV-CHN-2022-GXBSHJ0716-27	Guangxi, China	Jul 16, 2022	Canine	PP583099	PP583164	PP583229
CCoV-CHN-2022-GXBSHJ0723-28	Guangxi, China	Jul 23, 2022	Canine	PP583100	PP583165	PP583230
CCoV-CHN-2022-GXBSHJ0730-29	Guangxi, China	Jul 30, 2022	Canine	PP583101	PP583166	PP583231
CCoV-CHN-2023-GXBSHJ0221-30	Guangxi, China	Feb 21, 2023	Canine	PP583102	PP583167	PP583232
CCoV-CHN-2023-GXBSHM0213-31	Guangxi, China	Feb 13, 2023	Canine	PP583103	PP583168	PP583233
CCoV-CHN-2023-GXYLAC0304-32	Guangxi, China	Mar 4, 2023	Canine	PP583104	PP583169	PP583234
CCoV-CHN-2022-GXBSHM1224-33	Guangxi, China	Dec 24, 2022	Canine	PP583105	PP583170	PP583235
CCoV-CHN-2023-GXBSHM0328-34	Guangxi, China	Mar 28, 2023	Canine	PP583106	PP583171	PP583236
CCoV-CHN-2023-GXYLAC0318-35	Guangxi, China	Mar 28, 2023	Canine	PP583107	PP583172	PP583237
CCoV-CHN-2023-GXBSHJ0316-36	Guangxi, China	Mar 16, 2023	Canine	PP583108	PP583173	PP583238
CCoV-CHN-2023-GXBSHJ0328-37	Guangxi, China	Mar 28, 2023	Canine	PP583109	PP583174	PP583239
CCoV-CHN-2023-GXBSHM0413-38	Guangxi, China	Apr 13, 2023	Canine	PP583110	PP583175	PP583240
CCoV-CHN-2023-GXBSHM0518-39	Guangxi, China	May 18, 2023	Canine	PP583111	PP583176	PP583241
CCoV-CHN-2023-GXBSHJ0520-40	Guangxi, China	May 20, 2023	Canine	PP583112	PP583177	PP583242
CCoV-CHN-2023-GXYLAC0503-41	Guangxi, China	May 3, 2023	Canine	PP583113	PP583178	PP583243
CCoV-CHN-2023-GXQZSM0601-42	Guangxi, China	Jun 1, 2023	Canine	PP583114	PP583179	PP583244
CCoV-CHN-2023-GXGLKB0529-43	Guangxi, China	May 28, 2023	Canine	PP583115	PP583180	PP583245
CCoV-CHN-2023-GXBSHM0529-44	Guangxi, China	May 29, 2023	Canine	PP583116	PP583181	PP583246
CCoV-CHN-2023-GXBSHM0616-45	Guangxi, China	Jun 16, 2023	Canine	PP583117	PP583182	PP583247
CCoV-CHN-2023-GXBSHJ0703-46	Guangxi, China	Jul 3, 2023	Canine	PP583118	PP583183	PP583248
CCoV-CHN-2023-GXNNAY0711-47	Guangxi, China	Jul 11, 2023	Canine	PP583119	PP583184	PP583249
CCoV-CHN-2023-GXBSHJ0802-48	Guangxi, China	Aug 2, 2023	Canine	PP583120	PP583185	PP583250
CCoV-CHN-2023-GXBSHM0815-49	Guangxi, China	Aug 15, 2023	Canine	PP583121	PP583186	PP583251
CCoV-CHN-2023-GXYLAC0903-50	Guangxi, China	Sep 3, 2023	Canine	PP583122	PP583187	PP583252
CCoV-CHN-2022-GXLZBD0310-51	Guangxi, China	Mar 10, 2022	Canine	PP583123	PP583188	PP583253
CCoV-CHN-2022-GXLZBD0415-52	Guangxi, China	Apr 15, 2022	Canine	PP583124	PP583189	PP583254
CCoV-CHN-2023-GXYLAC1001-53	Guangxi, China	Oct 1, 2023	Canine	PP583125	PP583190	PP583255
CCoV-CHN-2022-GXYLAC0702-54	Guangxi, China	Jul 2, 2022	Canine	PP583126	PP583191	PP583256
CCoV-CHN-2023-GXBSHM1106-55	Guangxi, China	Nov 6, 2023	Canine	PP583127	PP583192	PP583257
CCoV-CHN-2023-GXBSHM1205-56	Guangxi, China	Dec 5, 2023	Canine	PP583128	PP583193	PP583258
CCoV-CHN-2023-GXNNAY1206-57	Guangxi, China	Dec 6, 2023	Canine	PP583129	PP583194	PP583259
CCoV-CHN-2023-GXNNAY1206-58	Guangxi, China	Dec 6, 2023	Canine	PP583130	PP583195	PP583260
CCoV-CHN-2023-GXNNAY1213-59	Guangxi, China	Dec 13, 2023	Canine	PP583131	PP583196	PP583261
CCoV-CHN-2022-GXNNCL0717-60	Guangxi, China	Jul 17, 2022	Canine	PP583132	PP583197	PP583262
CCoV-CHN-2022-GXNNCL0509-61	Guangxi, China	May 9, 2022	Canine	PP583133	PP583198	PP583263
CCoV-CHN-2024-GXBSHM0108-62	Guangxi, China	Jan 8, 2024	Canine	PP583134	PP583199	PP583264
CCoV-CHN-2024-GXBSHJ0205-63	Guangxi, China	Feb 5, 2024	Canine	PP583135	PP583200	PP583265
CCoV-CHN-2024-GXBSHM0313-64	Guangxi, China	Mar 13, 2024	Canine	PP583136	PP583201	PP583266
CCoV-CHN-2024-GXLZXL0227-65	Guangxi, China	Feb 27, 2024	Canine	PP583137	PP583202	PP583267

Table S3 Information on CCoV, FCoV, and TGEV Used for Phylogenetic Analysis

Strain	Location	Code Letter	Date	Host	Gene Accession Number	genotype
					S / M / N	
CCoV/NC0520	China	CHN	2020	Canine	MZ173443, S gene CDS	I
CCoV Elmo/02	Italy	ITA	2002	Canine	AY307020, S gene CDS	I
CCoV 23/03	Italy	ITA	2002	Canine	AY307021, S gene CDS	I
SWU-SSX3/2021/CCoVI	China	CHN	Jan, 2021	Canine	OK340206, S gene CDS	I
SWU-SSX2/2021/CCoVI	China	CHN	Jan, 2021	Canine	OK340205, S gene CDS	I
SWU-SSX1/2021/CCoVI	China	CHN	Jan, 2021	Canine	OK340204, S gene CDS	I
SWU-SSX10/2021/CCoVI	China	CHN	May, 2021	Canine	OK340213, S gene CDS	I
CCoV B906_ZJ_2019	China	CHN	Dec, 2019	Canine	MT114554, S gene CDS	IIv
CCoV DM95/2003	China	CHN	2003	Canine	EF192156, S gene CDS	IIb
SWU-SSX7/2021/CCoVIIb	China	CHN	Jun, 2020	Canine	OK340210, S gene CDS	IIb
RDCoV/GZ43/2003	China	CHN	2003	Raccoon dog	EF192155, S gene CDS	IIa
SWU-SSX9/2021/CCoVIIa	China	CHN	Jun, 2020	Canine	OK340212, S gene CDS	IIa
SWU-SSX6/2021/CCoVIIa	China	CHN	Jun, 2020	Canine	OK340209, S gene CDS	IIa
SWU-SSX5/2021/CCoVIIa	China	CHN	Jun, 2020	Canine	OK340208, S gene CDS	IIa
SWU-SSX4/2021/CCoVIIa	China	CHN	Jun, 2021	Canine	OK340207, S gene CDS	IIa
SWU-SSX8/2021/CCoVIIa	China	CHN	Jun, 2020	Canine	OK340211, S gene CDS	IIa
CCoV B617_ZJ_2019	China	CHN	Sep, 2019	Canine	MT114553 S gene CDS	IIa
CCoV B001_AH_2018	China	CHN	Nov, 2019	Canine	MT114552, S gene CDS	IIa
CCoV B020_HLJ_2018	China	CHN	Sep, 2018	Canine	MT114551, S gene CDS	IIa
CCoV B157_HLJ_2019	China	CHN	Jan, 2019	Canine	MT114550, S gene CDS	IIa
CCoV B825_ZJ_2019	China	CHN	Sep, 2019	Canine	MT114549, S gene CDS	IIa
CCoV B179_GZ_2019	China	CHN	Jan, 2019	Canine	MT114548, S gene CDS	IIa
CCoV B858_ZJ_2019	China	CHN	Nov, 2019	Canine	MT114547, S gene CDS	IIa
CCoV B793_ZJ_2019	China	CHN	Nov, 2019	Canine	MT114546, S gene CDS	IIa
CCoV B795_ZJ_2019	China	CHN	Nov, 2019	Canine	MT114545, S gene CDS	IIa
CCoV C21111102	China	CHN	Nov, 2021	Canine	OQ351916, S gene CDS	IIa
CCoV C21110201	China	CHN	Nov, 2021	Canine	OQ351915, S gene CDS	IIa
CCoV C21041821-2	China	CHN	Apr, 2021	Canine	OQ351914, S gene CDS	IIa
CCoV C21032451-1	China	CHN	Mar, 2021	Canine	OQ351913, S gene CDS	IIa
CCoV/dog/HCM27/2014	Viet Nam	VNM	Jan, 2014	Canine	LC190906, S gene CDS	IIa
CCoV PVNRTVU/2020-0001	India	IN	2020	Canine	MT955604, S gene CDS	IIa
CCoV giant panda strain	China	CHN	2003	giant panda	AY436637, S gene CDS	IIa
CCV-S-QN-30	China	CHN	Mar, 2018	Canine	MT166676, S gene CDS	IIa
CCV-S-QN-3	China	CHN	Mar, 2018	Canine	MT166674, S gene CDS	IIa
CCoV v1	China	CHN	2003	Canine	AY390342, S gene CDS	IIa
CCoV SD-MO5	China	CHN	2021	Canine	OM864519, S gene CDS	IIa
CCoV 259/01	Italy	ITA	2002	Canine	AF502583, M gene CDS	FCoV-like IIa
CCoV giant panda strain	China	CHN	2003	giant panda	AY436635, M gene CDS	IIa
CCoV v1	China	CHN	2003	Canine	AY390343, M gene CDS	IIa
CCoVNv2	China	CHN	2003	Canine	AY390344, M gene CDS	IIa
CCoV HF3	China	CHN	2004	healthy fox	AY864661, M gene CDS	IIa
CCoV HR	China	CHN	2005	Canine	AY884049, M gene CDS	IIa
CCoV HC2	China	CHN	2005	Canine	AY884048, M gene CDS	IIa
CCoV 04-0709	United Kingdom	UK	2008	Canine	FJ009114, M gene CDS	IIa
CCoV 04-0377	United Kingdom	UK	2008	Canine	FJ009115, M gene CDS	IIa
CCoV JS1706	China: jiangsu	CHN	2017	Canine	MN078152, M gene CDS	IIa
CCoV JS1712	China: jiangsu	CHN	2017	Canine	MN078151, M gene CDS	IIa
CCoV GM-26	China	CHN	Mar, 2018	Canine	MT136088, M gene CDS	IIa
CCoV DF	China	CHN	2004	fox	AY864662, M gene CDS	IIa
CCoV/SN0623	China	CHN	2020	Canine	MW718818, M gene CDS	IIa
CCoV/CD0529	China	CHN	2020	Canine	MW718815, M gene CDS	IIa
CCoV/GY0609	China	CHN	2020	Canine	MW718812, M gene CDS	IIa
CCoV/GY0529	China	CHN	2020	Canine	MW718810, M gene CDS	IIa
CCoV/NC0520	China	CHN	2020	Canine	MW718807, M gene CDS	IIa
CCoV tn449	USA	USA	1980	Canine	AY899209, M gene CDS	IIa
CCV NJ17	China	CHN	2004	Canine	AY704917, M gene CDS	IIa
CCoV A10	China	CHN	Mar, 2018	Canine	MT136097, M gene CDS	IIa
CCoV B1	China	CHN	Mar, 2018	Canine	MT136094, M gene CDS	IIa
CCoV BL-11	China	CHN	Mar, 2018	Canine	MT136090, M gene CDS	IIa
CCoV A2	China	CHN	Mar, 2018	Canine	MT136103, M gene CDS	IIa
CCoV C2	China	CHN	Mar, 2018	Canine	MT136081, M gene CDS	IIa
CCoV GM-23	China	CHN	Mar, 2018	Canine	MT136076, M gene CDS	IIa
CCoV NS-26	China	CHN	Mar, 2018	Canine	MT136075, M gene CDS	IIa
CCoV NS-22	China	CHN	Mar, 2018	Canine	MT136065, M gene CDS	IIa
CCoV LL-21	China	CHN	Mar, 2018	Canine	MT136070, M gene CDS	IIa
CCoV QN-2	China	CHN	Mar, 2018	Canine	MT136060, M gene CDS	IIa
CCoV v1	China	CHN	2003	Canine	AY390345, N gene CDS	IIa
CCoV v2	China	CHN	2003	Canine	AY390346, N gene CDS	IIa
CCoV giant panda strain	China	CHN	2003	giant panda	AY436636, N gene CDS	IIa

CCoV TN449	China	CHN	2006	Canine	EF056485, N gene CDS	IIa
CCoV/SN0623	China	CHN	2020	Canine	MW718806, N gene CDS	IIa
CCoV/CD0605	China	CHN	2020	Canine	MW718804, N gene CDS	IIa
CCoV/DZ0623	China	CHN	2020	Canine	MW718797, N gene CDS	IIa
CCoV/GY0609	China	CHN	2020	Canine	MW718800, N gene CDS	IIa
CCoV/DZ0623	China	CHN	2020	Canine	MW718797, N gene CDS	IIa
CCoV JS1706	China	CHN	Jun, 2017	Canine	MN163040, N gene CDS	IIa
CCoV JS1712	China	CHN	Dec, 2017	Canine	MN163039, N gene CDS	IIa
CCoV fc00-089	Japan	JPN	2013	Canine	AB781805, N gene CDS	IIa
CCoV fc94-039	Japan	JPN	2013	Canine	AB781802, N gene CDS	IIa
CCoV fc4	Japan	JPN	2013	Canine	AB781797, N gene CDS	IIa
CCoV fc76	Japan	JPN	2013	Canine	AB781800, N gene CDS	IIa
CCoV-BJ70	China	CHN	Jan, 2014	Canine	KT852997, N gene CDS	IIa
CCoV CFBCoV/DM95/2003	China	CHN	2003	Canine	EF192158, N gene CDS	IIa
CCoV RDCoV/GZ43/2003	China	CHN	2003	Canine	EF192157, N gene CDS	IIa
CCoV 23/03	Italy	ITA	2004	Canine	AY548235, M N gene CDS	I
CCoV CD37_21032456	China	CHN	Mar, 2021	Canine	OQ623733, M N gene CDS	I
CCoV CD28_21032427	China	CHN	May, 2021	Canine	OQ623732, M N gene CDS	I
CCoV CD5_20111930	China	CHN	Nov, 2020	Canine	OQ623730, M N gene CDS	I
CCoV CD1_20111203	China	CHN	Nov, 2020	Canine	OQ623729, M N gene CDS	I
CCoV CD38_21041814	China	CHN	Apr, 2021	Canine	OQ623734, M N gene CDS	I
CCoV CD21_21012004	China	CHN	Jan, 2021	Canine	OQ623731, M N gene CDS	I
CCoV CD23_21012019	China	CHN	Jan, 2020	Canine	OQ623723, M N gene CDS	IIa
CCoV CD15_20122320	China	CHN	Dec, 2020	Canine	OQ623719, M N gene CDS	IIa
CCoV CD6_20120106	China	CHN	Dec, 2020	Canine	OQ623715, M N gene CDS	IIa
CCoV CD2_20111206	China	CHN	Nov, 2020	Canine	OQ623713, M N gene CDS	IIa
CCoV CD30_21032441	China	CHN	Mar, 2021	Canine	OQ623726, M N gene CDS	IIa
CCoV CD34_21032452	China	CHN	Mar, 2021	Canine	OQ623695, M N gene CDS	IIa
CCoV CD26_21032414	China	CHN	Mar, 2021	Canine	OQ623694, M N gene CDS	IIa
CCoV CD44_21052023	China	CHN	Mar, 2021	Canine	OQ623728, M N gene CDS	IIa
CCoV CD4_20111923	China	CHN	Nov, 2020	Canine	OQ623714, M N gene CDS	IIa
CCoV 23/03	Italy	ITA	2003	Canine	KP849472, complete genome	I
CCoV 10/22	United Kingdom	UK	2022	Canine	OX335534, complete genome	I
CCoV 61/22	United Kingdom	UK	2022	Canine	OX335623, complete genome	I
CCoV 11/22	United Kingdom	UK	2022	Canine	OX335541, complete genome	I
CCoV 12/20	United Kingdom	UK	2022	Canine	OX335549, complete genome	I
CCoV 2020/15	United Kingdom	UK	2020	Canine	MT906864, complete genome	IIv
CCoV A76	USA: Ithaca, NY	USA	1976	Canine	JN856008, complete genome	IIv
CCoV 430/07	Italy	ITA	Oct, 2007	Canine	EU924790, complete genome	IIb
CCoV 119/08	Italy	ITA	Mar, 2008	Canine	EU924791, complete genome	IIb
CCoV 174/06	Italy	ITA	Mar, 2006	Canine	EU856362, complete genome	IIb
CCoV 341/05	Italy	ITA	Dec, 2005	Canine	EU856361, complete genome	IIb
CCoV 66/09	Greece	GRC	2009	Canine	HQ450376, complete genome	IIb
CCoV 68/09	Greece	GRC	2009	Canine	HQ450377, complete genome	IIb
CCoV-HuPn-2018	Malaysia	MAS	2017	Homo sapiens	MW591993, complete genome	IIb
CCoV Z19	Haiti	HTI	2017	Homo sapiens	MZ420153, complete genome	IIb
CCoV/7/2020/AUS	Australia	AUS	Mar, 2020	Canine	MW383487, complete genome	IIb
CCoV/dog/HCM47/2015	Viet Nam	VNM	Jun, 2015	Canine	LC190907, complete genome	IIb
CCoV/NTU336/F/2008	China Taiwan	CHN_TW	Nov, 2008	Canine	GQ477367, complete genome	IIb
CCoV 450/07	Italy	ITA	Oct, 2007	Canine	GU146061, complete genome	IIb
CCoV BM35	China	CHN	Nov, 2019	Canine	MT919267, complete genome	IIa
CCoV JS2103	China	CHN	Mar, 2021	Canine	OM055788, complete genome	IIa
CCoV 41	China	CHN	Nov, 2019	Fox	MW354911, S M N gene CDS	IIa
CCoV 38	China	CHN	Nov, 2019	Fox	MW354910, S M N gene CDS	IIa
CCoV/GD/2020/X9	China	CHN	Feb, 2020	Canine	MZ320954, complete genome	IIa
CCoV/GD/2020/X8	China	CHN	Feb, 2020	Canine	MZ320953, complete genome	IIa
CCoV/PK02/2019/BRA	Brazil	BR	2019	Canine	OP179857, complete genome	IIa
CCoV 1-71	Germany	GER	1972	Canine	JQ404409, complete genome	IIa
CCoV TN-449	USA	USA	1980	Canine	JQ404410, complete genome	IIa
CCoV K378	USA	USA	1978	Canine	KC175340, complete genome	IIa
CCoV INSAVC-1	UK	UK	1992	Canine	D13096, complete genome	IIa
CCoV BGF10	United Kingdom	UK	2002	Canine	AY342160, complete genome	IIa
CCoV CB/05	Italy	ITA	2005	Canine	KP981644, complete genome	IIa
CCoV 171	Germany	GER	1971	Canine	KC175339, complete genome	IIa
CCoV B135_JS_2018	China	CHN	Oct, 2019	cloacal swab	MT114544, complete genome	IIa
CCoV B194_GZ_2019	China	CHN	Jan, 2019	cloacal swab	MT114543, complete genome	IIa
CCoV B203_GZ_2019	China	CHN	Jan, 2019	cloacal swab	MT114542, complete genome	IIa
CCoV B363_ZJ_2019	China	CHN	Jul, 2019	cloacal swab	MT114541, complete genome	IIa
CCoV B447_ZJ_2019	China	CHN	Jul, 2019	cloacal swab	MT114540, complete genome	IIa
CCoV B600_ZJ_2019	China	CHN	Sep, 2019	cloacal swab	MT114539, complete genome	IIa
CCoV B639_ZJ_2019	China	CHN	Sep, 2019	cloacal swab	MT114538, complete genome	IIa
CCoV SH110_2007	Tanzania	TZA	2007	Crocuta crocuta	MF095850, complete genome	IIa
CCoV SH33_2007	Tanzania	TZA	2007	Crocuta crocuta	MF095849, complete genome	IIa

CCoV S378	USA: Ithaca, NY	USA	1978	Canine	KC175341, complete genome	IIa
CCoV fc1	Japan	JPN	1990	Canine	AB781790, complete genome	IIa
CCoV SH32_2001	Tanzania	TZA	2001	Crocuta crocuta	MF095847, complete genome	IIa
CCoV NA/09	Greece	GRC	2009	Canine	JF682842, complete genome	IIa
CCoV SBJ12_2007	Tanzania	TZA	2007	Canis mesomelas	MF095854, complete genome	IIa
CCoV SBJ3_2011	Tanzania	TZA	2011	Crocuta crocuta	MF095855, complete genome	IIa
CCoV SH157_2012	Tanzania	TZA	2012	Crocuta crocuta	MF095853, complete genome	IIa
CCoV SH89_2011	Tanzania	TZA	2011	Crocuta crocuta	MF095851, complete genome	IIa
CCoV SH143_2011	Tanzania	TZA	2011	Crocuta crocuta	MF095852, complete genome	IIa
CCoV SH36_2004	Tanzania	TZA	2004	Crocuta crocuta	MF095848, complete genome	IIa
CCoV 447_ZJ_201	China	CHN	Jul, 2019	Canine	MT114540, complete genome	IIa
VuCCoV_191140SY	China	CHN	Nov, 2019	Canine	OQ540915, complete genome	IIa
VuCCoV_191109SY	China	CHN	Nov, 2019	Canine	OQ540922, complete genome	IIa
VuCCoV_191108SY	China	CHN	Nov, 2019	Canine	OQ540921, complete genome	IIa
VuCCoV_191107SY	China	CHN	Nov, 2019	Canine	OQ540920, complete genome	IIa
VuCCoV_191128SY	China	CHN	Nov, 2019	Canine	OQ540909, complete genome	IIa
VuCCoV_191118SY	China	CHN	Nov, 2019	Canine	OQ540913, complete genome	IIa
CCoV SMU-8	China	CHN	Jan, 2020	Canine	ON107244, complete genome	IIa
CCoV CB/05	Italy	ITA	2005	Canine	DQ112226, complete genome	IIa
CCoV GH4-2	China	CHN	2020	Canine	OM950729, complete genome	IIa
CCoV GH8-2	China	CHN	2020	Canine	OM950728, complete genome	IIa
CCoV SD-A1	China	CHN	2021	Canine	OM864518, S M N gene CDS	IIa
CCoV HeB-G1	China	CHN	2021	Canine	OM451123, complete genome	IIa
CCoV SD-F3	China	CHN	2021	Canine	OM451122, complete genome	IIa
CCoV 2020/7	United Kingdom	UK	2020	Canine	MT906865, complete genome	IIa
CCoV JS1712	China	CHN	Dec, 2017	Canine	MW186441, complete genome	IIa
CCoV JS1706	China	CHN	Jun, 2017	Canine	MW186440, complete genome	IIa
CCoV HLJ-073	China	CHN	Jul, 2016	Canine	KY063618, complete genome	FCoV2 IIa
CCoV HLJ-072	China	CHN	Jul, 2016	Canine	KY063617, complete genome	FCoV2 IIa
CCoV HLJ-071	China	CHN	Jul, 2016	Canine	KY063616, complete genome	FCoV2 IIa
FCoV/CD0524	China	CHN	2020	Feline	MW815657, S gene CDS	I
FIPV UCD1	USA	USA	1976	Feline	AB088222, S gene CDS	I
FIPV HRB/XF17	China	CHN	Jul, 2017	Feline	MK987175, S gene CDS	II
FCoV/China/SMU-CD59/2018	China	CHN	Oct, 2018	Feline	MW316851, S gene CDS	II
FCoV UU4	The Netherlands	NED	Feb, 2007	Feline	FJ938054, complete genome	I
FCoV UU20	The Netherlands	NED	Aug, 2007	Feline	HQ392471, complete genome	I
FCoV UU31	The Netherlands	NED	Jan, 2008	Feline	HQ012371, complete genome	I
FCoV QS	China	CHN	May, 2018	Feline	MW030108, complete genome	I
FCoV HLJ/DQ/2016/01	China	CHN	Oct, 2016	Feline	KY292377, complete genome	I
FCoV UU11	The Netherlands	NED	Jun, 2007	Feline	FJ938052, complete genome	I
FCoV FIPV-UCD11a	USA	USA	Jan, 2008	Feline	FJ917519, complete genome	I
FCoV UU54	The Netherlands	NED	Mar, 2010	Feline	JN183883, complete genome	I
FCoV TCVSP-ROTTIER-00023	The Netherlands	NED	Sep, 2007	Feline	GU553362, complete genome	I
FCoV 26M	United Kingdom	UK	Jan, 2013	Feline	KP143512, complete genome	I
FCoV RM	USA: California	USA	Jan, 2002	Feline	FJ938051, complete genome	I
FCoV UU88	The Netherlands	NED	Aug, 2010	Feline	KF530123, complete genome	I
FCoV HF1902	China	CHN	May, 2019	Feline	MT444152, complete genome	I
FCoV XXN	China	CHN	Apr, 2018	Feline	MN165107, complete genome	I
FCoV UG-FH8	Belgium	BEL	Jan, 2015	Feline	KX722529, complete genome	I
FCoV C1Je	United Kingdom	UK	2006	Feline	DQ848678, complete genome	I
FCoV inoculum	Belgium	BEL	2013	Feline	KU215419, complete genome	I
FCoV FIPV Cat 1 Karlslunde	Denmark	DEN	Feb, 2015	Feline	KX722530, complete genome	I
FCoV VP1a	Germany: Kassel	GER	2006	Feline	MW308128, complete genome	I
FCoV Black	USA	USA	1970	Feline	EU186072, complete genome	I
FCoV FPV-1	Australia	AUS	2017	Feline	ON595853, complete genome	I
FCoV Felix	Germany	GER	Jul, 2012	Feline	MG893511, complete genome	I
FCoV M91-267	Japan	JPN	1991	Feline	AB781788, complete genome	II
FCoV KUK-H/L	Japan	JPN	1987	Feline	AB781789, complete genome	II
FIPV 79-1146	United States	USA	1979	Feline	AY994055, complete genome	II
FCoV Tokyo/cat/130627	Japan: Tokyo	JPN	2013	Feline	AB907624, complete genome	II
FCoV WSU 79-1683	USA	USA	2011	Feline	JN634064, complete genome	II
FIPV DF-2	United States	USA	1980	Feline	JQ408981, complete genome	II
FCoV/NTU156/P	China Taiwan	CHN_TW	Sep, 2007	Feline	GQ152141, complete genome	II
TGEV TH-98	China	CHN	2002	porcine	AF494337, S gene CDS	/
TGEV Vaccine 462	Russia	RU	Sep, 1972	porcine	ON324116, S gene CDS	/
TGEV TO14	Japan	JPN	2000	porcine	AF302262, M gene CDS	/
TGEV KT3	South Korea	SKO	2012	porcine	JQ693056, M gene CDS	/
TGEV/GanSu/2022/China	China	CHN	May, 2021	porcine	OM847397, N gene CDS	/
TGEV CH/HLJD/09	China	CHN	2009	porcine	GQ374558, N gene CDS	/
TGEV WH-1	China	CHN	2010	Sus scrofa	HQ462571, complete genome	/
TGEV Purdue P115	USA	USA	2006	porcine	DQ811788, complete genome	/
TGEV 96-1933	United Kingdom	UK	1933	porcine	AF104420, complete genome	/
TGEV virulent Purdue	USA: Indiana	USA	1952	porcine	DQ811789, complete genome	/
TGEV AHHF	China	CHN	Dec, 2015	porcine	KX499468, complete genome	/
TGEV/USA/SouthDakota154/2014	USA: South Dakota	USA	Feb, 2014	porcine	KX900411, complete genome	/
